# On the Shortening of the Lead Time of Ocean Warm Water Volume to ENSO SST Since 2000

**DOI:** 10.1038/s41598-017-04566-z

**Published:** 2017-06-27

**Authors:** Zeng-Zhen Hu, Arun Kumar, Jieshun Zhu, Bohua Huang, Yu-heng Tseng, Xiaochun Wang

**Affiliations:** 1 Climate Prediction Center, NCEP/NWS/NOAA 5830 University Research Court, College Park, MD 20740 USA; 2 Earth System Science Interdisciplinary Center, the University of Maryland, College Park, Maryland 20740 USA; 3 Department of Atmospheric, Oceanic, and Earth Sciences and Center for Ocean–Land–Atmosphere Studies George Mason University, 4400 University Drive, Fairfax, VA 22030 USA; 4 Institute of Oceanography, National Taiwan University No. 1, Sec. 4, Roosevelt Rd., Taipei, 10617 Taiwan; 5 School of Marine Sciences Nanjing University of Information Sciences and Technology, Nanjing, 210044 China; 6 JIFRESSE, University of California at Los Angeles, Los Angeles, CA 90095 USA

## Abstract

The possible factors associated with the shortening of lead time between ocean warm water volume (WWV) variability along the equatorial Pacific and El Niño–Southern Oscillation (ENSO) variability after 2000 are documented. It is shown that the shortening of lead time is due to frequency increases of both WWV and ENSO. During 1979–99 the dominant frequencies were 1.5–3.5 years for both the Niño3.4 and WWV indices. In contrast, during 2000–16, both indices had a relatively flatter spectrum and were closer to a white noise process with a relative maximum at 1.5–2.0 years for the Niño3.4 index and 0.8–1.3 years for the WWV index. The frequency change of ENSO and WWV were linked to a westward shift of the Bjerknes feedback region. The results here are consistent with previous argument that the westward shift of the air-sea coupling region will cause an increase of ENSO frequency, as the corresponding zonal advection feedback reduces the period and growth of coupled instability, thus favoring more frequent and weak El Niño events.

## Introduction

**Figure 1:**
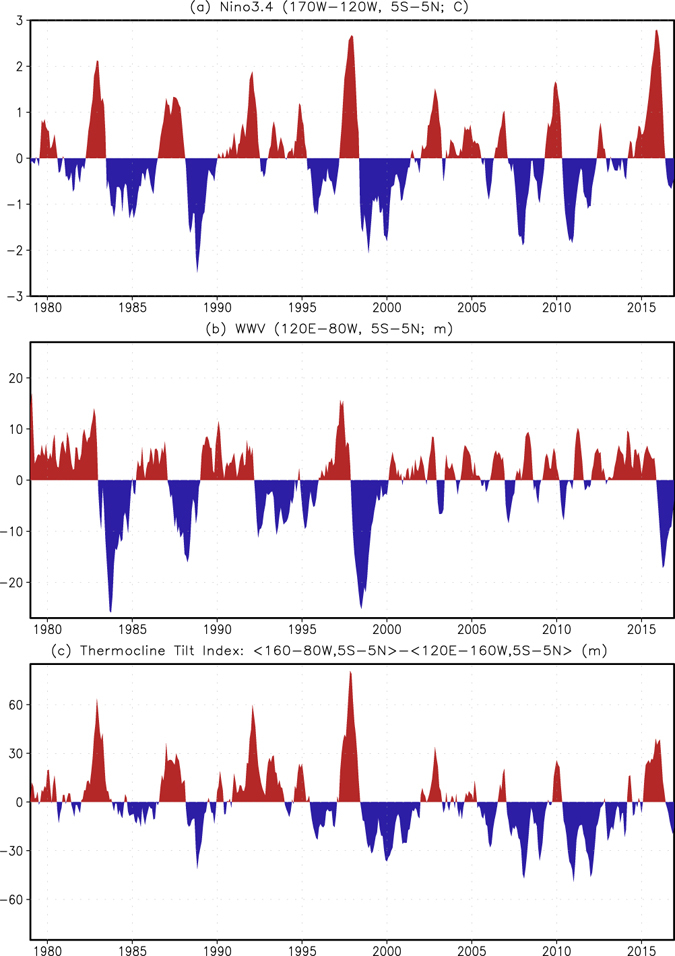
Time series of (**a**) Niño3.4, (**b**) WWV, and (**c**) tilt indices during Jan 1979-Dec 2016. Niño3.4 index is defined as SSTA averaged in (5°S–5°N, 120°–170°W), WWV index as D20 anomaly averaged in (5°S–5°N, 120°E–80°W), and tilt index as D20 anomaly difference between means in the east (5°S–5°N, 80°–160°W) and west (5°S–5°N, 120°E–160°W). Figure is generated by GrADS (http://cola.gmu.edu/grads/).

The El Niño–Southern Oscillation (ENSO) dominates the tropical Pacific climate variability at interseasonal to interannual time scales and is also the major source of predictability of global climate variability at these time scales^1–3^. ENSO forecasts have now become operational at several centers^4, 5^. ENSO prediction skill, however, has not demonstrated a steady improvement during the past few decades. For example, Wang *et al*.^2^, Barnston *et al*.^5^ and Kumar *et al*.^6^ noted a decrease of the prediction skill (measured by linear correlation coefficient) after 1999/2000. The prediction skill relies on both the forecast tools (such as, climate models and statistical techniques), and the observations that initialize forecasts, as well as the predictand, e.g., ENSO. With the progress in model development and in the observing system with time, the decline of the prediction skill, therefore, should mainly be due to the changes in the characteristics of ENSO. For instance, after 2000, ENSO variability decreased^7–9^, which is also linked to a reduction of subsurface ocean temperature variability or the strength of air-sea coupling of the whole tropical Pacific climate system^10, 11^ (Fig. 1). For instance, compared with those in 1982/83 and 1997/98 El Niños, both the amplitudes of the warm water volume (WWV) and tilt indices in 2015/16 El Niño are much smaller (Fig. 1b,c), although they have comparable amplitudes of sea surface temperature anomaly (SSTA) in the central and eastern equatorial Pacific (Fig. 1a).

**Figure 2:**
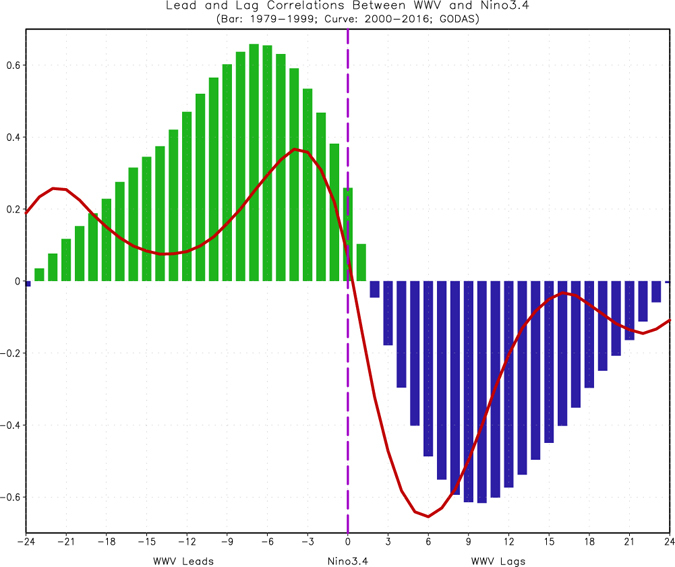
Lead and lag correlations between the Niño3.4 and WWV indices in Jan 1979–Dec 1999 (bar), and Jan 2000–Dec 2016 (curve). The negative (positive) numbers in the x-axis represent the number of months that the WWV index leads (lags) the Niño3.4 index. Figure is generated by GrADS (http://cola.gmu.edu/grads/).

The decrease of ENSO prediction skill may also be associated with a breakdown of the relationship between WWV integrated along the equatorial Pacific and ENSO SSTAs^7^. For example, WWV led ENSO SSTAs by 6–8 months during 1979–99 (bars in Fig. 2), while the WWV variations decreased and lead time also reduced to only 3–4 months during 2000–16 (curve in Fig. 2), as had been indicated by McPhaden^7^, Horii *et al*.^12^, Kumar and Hu^10^, Hu *et al*.^9^.

It is unclear why the relationship between WWV and ENSO SSTAs deteriorated in recent decades. Both McPhaden^7^, Horii *et al*.^12^, and Lübbecke and McPhaden^13^ speculated that the breakdown may be connected with a shift towards more central Pacific versus eastern Pacific El Niños in the past decade (see Supplementary Fig. S1) which may also lead to changes in the relative importance of feedback mechanisms associated with ENSO variability. According to An and Wang^14^, ENSO frequency is modulated by the spatial structure of ENSO mode. Eastward (westward) shift of zonal wind stress anomaly with respect to SSTA during 1980–93 (1962–75) coincided with ENSO period increases (decreases). In this work, following An and Wang^14^, we compare the zonal location change of air-sea coupling during 1979–99 and 2000–16 to investigate the factors that result in ENSO and WWV frequency change as well as the lead time change between the WWV and ENSO SSTA after 1999/2000.

## Results

**Figure 3:**
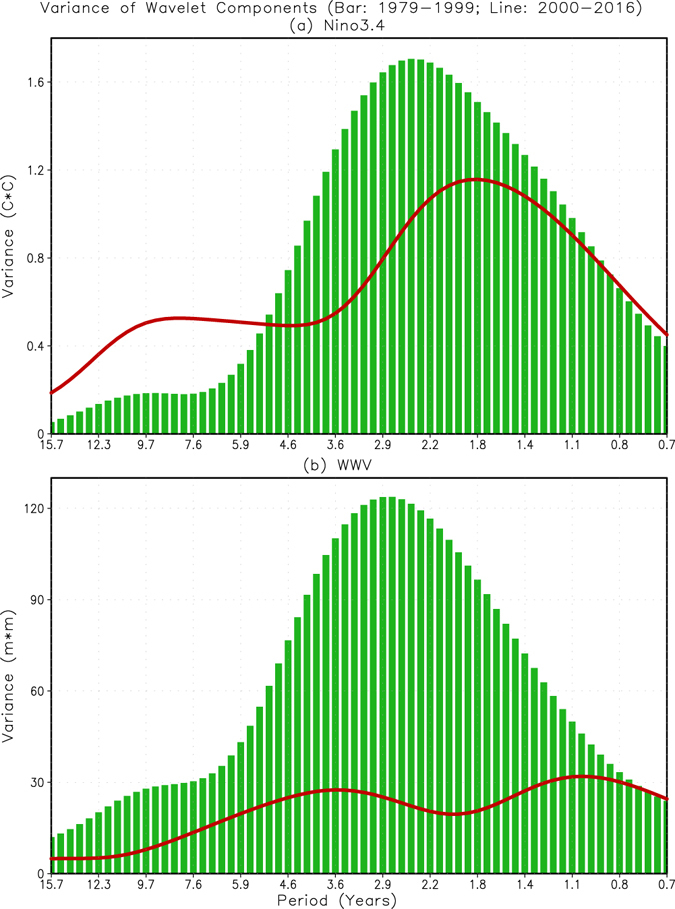
Variance dependence on time scales of the (**a**) Niño3.4 and (**b**) WWV indices for the average in Jan 1979–Dec 1999 (bar) and Jan 2000–Dec 2016 (curve), based on the time scale decomposition of wavelet. See text for the details of the calculation. Figure is generated by GrADS (http://cola.gmu.edu/grads/).

Through wavelet analysis^15, 16^, we first decompose variability in the Niño3.4 and WWV indices into different time scales, and then calculate the corresponding variances at various time scales. From Fig. 3, we see the shift in the variability from lower frequencies during 1979–99 (bar) to higher frequencies during 2000–16 (curve) for both indices, in addition to large reductions of the maximum variances. Specifically, during 1979–99 (bars in Fig. 3), the variances peak at time scales of 1.5–3.5 years for both the Niño3.4 and WWV indices. In contrast, during 2000–16, the variances don’t have an obvious peak, and only a relatively weak maximum presents in 1.5–2.0 years for the Niño3.4 index (curve in Fig. 3a) and 0.8–1.3 years for the WWV index (curve in Fig. 3b). Here, we should point out that the evolution of WWV index is a measurement of integrated equatorial ocean heat recharge and discharge processes^17^. The shift to a higher frequency for WWV index implies that the equatorial Pacific Ocean heat content fluctuated at a faster pace after 2000.

Comparing the variance levels between these two periods, there is almost no substantial WWV “energy” in 2000–16 (Fig. 3b). This is also the case for the variability of the tilt index [defined as difference in D20 between the eastern (5°S–5°N, 160°–80°W) and western (5°S–5°N, 120°E–160°W) equatorial Pacific; Supplementary Fig. S2], a measurement for the available potential energy of west-east oscillation of ocean temperature in the equatorial ocean^18^.

Thus, in addition to the frequency change of ENSO SSTA indicated in Hu *et al*.^11^, subsurface ocean heat also fluctuated at a relatively higher frequency after 2000, suggesting that ENSO and tropical Pacific climate variations shifted to a higher frequency regime. The shortening of the lead time of WWV to ENSO SSTA, therefore, is due to the shifting to higher frequencies for both WWV and ENSO SSTA after 2000. We note that compared to 1979–99, the variance distribution is flatter in 2000–16 for both the Niño3.4 and WWV indices (Fig. 3), implying that the variability of ENSO is closer to a white noise process during 2000–16, and thus, less predictable. Nevertheless, we should indicate that the analysis of ENSO frequency change after 2000 is based on short data period (Jan 1979–Dec 2016) and a few ENSO events, which may affect the robustness of the results to some extent.

What are potential causes for the frequency shift of both WWV and ENSO SSTA variabilities? According to An and Wang^14^, ENSO periodicity is affected by the spatial structure of ENSO associated surface wind stress and SSTAs. Through observational diagnoses and theoretical model experiments, they noted that eastward (westward) shift of the zonal wind stress with respect to the SSTAs results in an increase (a decrease) of ENSO period. They argued that when the zonal wind stress anomaly shifts westward, the corresponding zonal advection feedback reduces the period and growth of coupled instability, thus favoring more frequent and weaker El Niño events. As a result, ENSO events may not grow to large amplitude and decay early. A consequence is that ENSO amplitude decreases and its period shortens.

**Figure 4:**
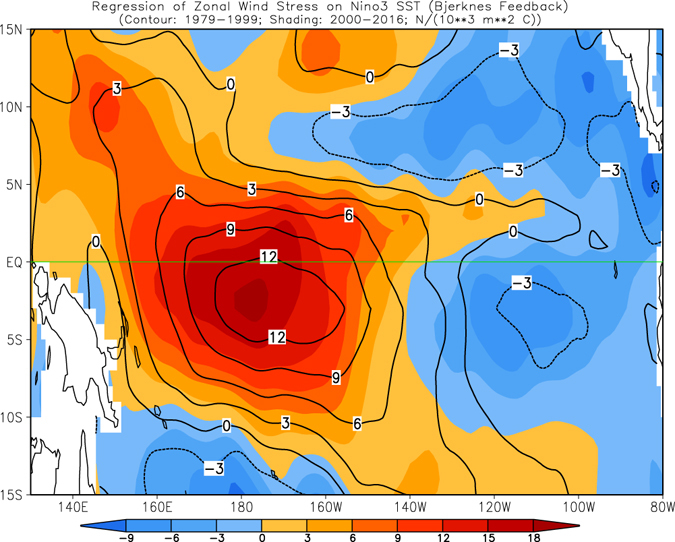
Simultaneous regressions of zonal wind stress anomalies onto the Niño3 index, which was referred to as the Bjerknes feedback. Contours (shadings) are for the regressions in Jan 1979–Dec 1999 (Jan 2000–Dec 2016). The unit is N/(10^3^ m^2^ °C) and the contour interval is 3. Figure is generated by GrADS (http://cola.gmu.edu/grads/).

During an ENSO development, air-sea coupling or so-called Bjerknes feedback plays a crucial role. Here, we compute the feedback, which is defined as the regressions of monthly mean zonal wind stress anomalies onto the Niño3 index following Lloyd *et al*.^19^. The regression coefficients represent the tropical ocean and atmosphere coupling strength. From Fig. 4, we note that the strong positive Bjerknes feedback is in the central and central-eastern tropical Pacific between 160°E and 145°W and extends to the northwestern Pacific during 1979–99 (contours). The maximum value center is in the Southern Hemisphere near (3°S, 170°W). In the southeastern tropical Pacific, the Bjerknes feedback is negative. The overall regression pattern shown in Fig. 4 is in agreement with that of Wittenberg *et al*.^20^ (see their Fig. 16). Also, the evolution of subsurface ocean temperature in these large positive Bjerknes feedback regions (175°E–155°W) has been proposed as a predictor for ENSO initiation and to monitor oceanic Kelvin wave propagation^21^.

**Figure 5:**
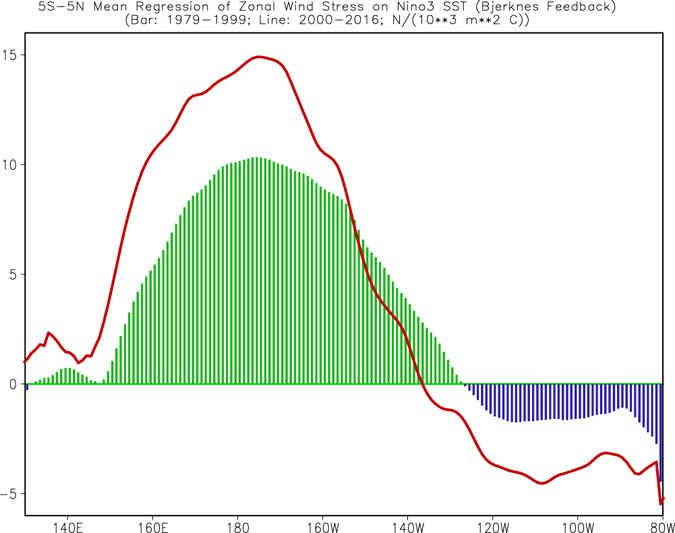
Same as Fig. 4, but for 5°S–5°N average in 1979–99 (bar) and 2000–16 (curve). Figure is generated by GrADS (http://cola.gmu.edu/grads/).

Compared with 1979–99 (contours, Fig. 4), the positive Bjerknes feedback after 2000 was strengthened in the western tropical and northwestern Pacific, but weakened in the region between 170°W and 115°W. On the other hand, the negative feedback was strengthened in the southeastern Pacific during 2000–16 (compare the shading and contours in Fig. 4). Such changes of the regressions suggest a northwestward shift of the air-sea coupling associated with the Bjerknes feedback. This shift is further confirmed by the average between 5°S–5°N (Fig. 5). In fact, the westward shift is seen in both Northern (0°–5°N) and Southern (5°S–0°) Hemispheres (Supplementary Fig. S3). For regressions onto Niño3.4 index (see Supplementary Fig. S4), overall regression pattern and their change from 1979–99 to 2000–16 are similar to corresponding regression onto Niño3 index (Fig. 4). In addition, for regressions onto Niño3 index with absolute value of Niño3 index less than 2 °C (see Supplementary Fig. S5), the results are also similar, implying that the westward shift of air-sea coupling from 1979–99 to 2000–16 is not dominated by a few extreme events and it is a robust result.

**Figure 6:**
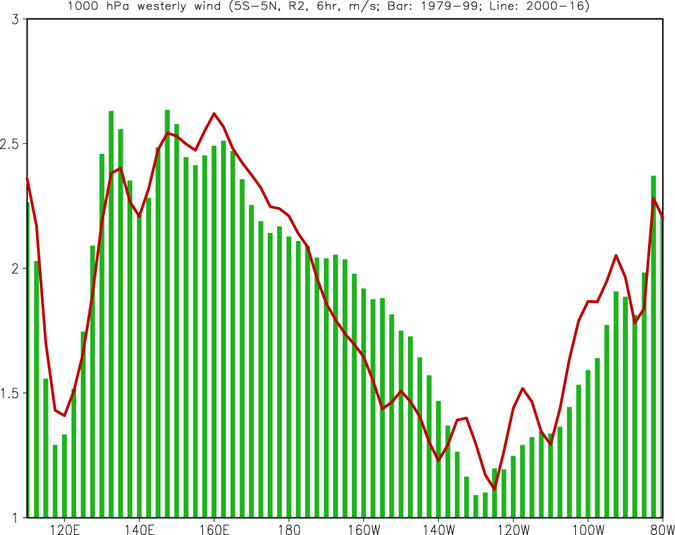
1000 hPa westerly zonal wind averaged in (5°S–5°N) and in Jan 1979–Dec 1999 (bar), and Jan 2000–Dec 2016 (line). The unit is m/s. The data are from R2 reanalysis and 6 hourly mean. The westerly zonal wind is referred to as u > 0. Figure is generated by GrADS (http://cola.gmu.edu/grads/).

Recently, Ding *et al*.^22^ argued that more Northern Hemisphere triggered El Niño (which favors occurrence of central Pacific type El Niño)^22^ presented after 2000, while more Southern Hemisphere triggered El Niño (eastern Pacific type El Niño) occurred before 2000. Hu *et al*.^23^ suggested that stronger and more eastward extended westerly wind bursts along the equatorial Pacific in early months of a year (Jan–Jul) are linked with active air–sea interaction over the cold tongue/the Intertropical Convergence Zone (ITCZ) complex, as well as more intensive oceanic thermocline feedback, favoring the eastern Pacific El Niño. Meanwhile, weaker and westward confined westerly wind bursts along the equatorial Pacific in early months of a year (Jan–Jul) are linked with suppressed air–sea interaction over the cold tongue/ITCZ complex, as well as less intensive oceanic thermocline feedback, favoring the central Pacific El Niño. Consistently, 1000 hPa westerly wind in the central and eastern equatorial Pacific Ocean (175°–140°W) decreases during 2000–16 compared with that in 1979–99 (Fig. 6), implying less eastward extension of westerly wind burst event after 2000. Such westward confinement of air-sea coupling after 2000 is visible from time and longitude evolution of D20, SST, and zonal wind stress anomalies along the equator (see Supplementary Fig. S1).

## Discussion

In this work, we investigated the possible factors associated with the shortening of lead time between warm water volume (WWV) along the equatorial Pacific and ENSO after 2000. It is noted that the shortening of lead time is coincided with the frequency increase of both WWV and ENSO. During 1979–99, the dominant frequencies were 1.5–3.5 years for both the Niño3.4 and WWV indices. In contrast, during 2000–16, both indices show a relatively flatter spectral distribution and are closer to a white noise process with relative maximum spectrum in 1.5–2.0 years for the Niño3.4 index and 0.8–1.3 years for the WWV index. The frequency change of ENSO and WWV were also reflected in the zonal shift of the Bjerknes feedback or air-sea coupling. Compared with 1979–99, both the positive Bjerknes feedback in the western tropical and northwestern Pacific, and the negative feedback in the southeastern tropical Pacific strengthened after 2000. Such changes of the air-sea coupling suggest a westward shift of the air-sea coupling associated with the Bjerknes feedback or ENSO growth. According to An and Wang^14^, the westward shift of the air-sea coupling favors the increase of ENSO frequency. When zonal wind stress anomaly associated with SST variation shifts westward (eastward), the zonal advection feedback favors (doesn’t favor) transition of ENSO cycle from one phase to another rather than its growth. Consequently, the growth of the amplitude of ENSO events is constrained (enhanced) and decreases (increases) and their period becomes shorter (longer)^14^.

The westward shift of the Bjerknes feedback or air-sea coupling is consistent with Bunge and Clarke^24^. They noted that the decrease of WWV lead time to ENSO is related to a relative increase of the contribution of the tilt (east-west dipole) mode of subsurface ocean temperature variability (a mode linked to the peak phase of ENSO^10^) and decrease of the contribution of second empirical orthogonal function mode to WWV (recharge and discharge mode^10, 25^). Both pre-1973 and post-1998 periods with reduced lead time of WWV to ENSO were characterized by mean La Niña-like conditions, including westward displacement of the associated anomalous wind forcing (see Supplementary Fig. S1). Bunge and Clarke^24^ further indicated that the westward displacement of zonal winds post-1998 increased the tilt mode contribution and decreases the recharge mode contribution to the WWV. Furthermore, the westward shift of the Bjerknes feedback links with the increase of central Pacific El Niño in recent decade^23, 26, 27^ (see Supplementary Fig. S1a). Compared with eastern Pacific El Niño, central Pacific El Niño is connected with weaker and more westward confined westerly wind anomalies averaged along the equatorial Pacific in early months of a year (Jan–Jul) as well as weaker air-sea interaction over the cold tongue/the Intertropical Convergence Zone complex and less intensive oceanic thermocline feedback^23^.

The westward shift of the Bjerknes feedback may be connected with the interdecadal change of mean state in the tropical Pacific in the context of ENSO^8, 28^. For instance, Hu *et al*.^8^ noted increased SST zonal gradient, enhanced trade wind, strengthened Walker circulation, and steeper thermocline slope after 2000. Such mean state changes may be due to the fact that the canonical El Niño can’t fully develop in the eastern Pacific, and thus, ENSO-related tropical Pacific variability decreases. They argued that too large thermocline slope (and too strong wind stress) along the equator is unfavorable for ENSO growth^8^.

Recently, Ding *et al*.^22^ argued the relative importance of extratropical northern and southern atmosphere in various decades in influencing the interdecadal changes in ENSO characteristics. When favorable influence from both extratropical northern and southern atmosphere present simultaneously, strong ENSO warm events may occur (commonly in 1979–99 and 2015/16 El Niño). If only the Northern or Southern Hemisphere impact exists, mostly weak or lack of El Niño may emerge. Most ENSO warm events after 2000 resulted from the Northern Hemispheric impact^22^, consistent with the northwestward shift of major Bjerknes feedback region after 2000 indicated in this work. 2014/15 ENSO evolution is a good example. The anomalies in the Northern Hemisphere provided a favorable condition to initiate an El Niño from July 2014, but the Southern Hemispheric subtropical SST cooling forcing suppressed the development^29^. As a result, just a boardline El Niño occurred in 2014/15 winter. This association is also consistent with previous works. For instance, with El Niños in 1980–2000, Kug *et al*.^30^ argued that the initiation of an El Niño by northern hemisphere winds was a common feature.

## Data and Methods

The ocean dataset analyzed in this work is from the Global Ocean Data Assimilation System (GODAS)^31^. Using the ocean temperature and depth of 20 °C isotherm (D20) from GODAS, the WWV and thermocline tilt indices were calculated. The WWV index is defined as an average of D20 between 120°E–80°W and 5°S–5°N^17^. The tilt index is defined as D20 anomaly difference between the east (5°S–5°N, 80°–160°W) and the west (5°S–5°N, 120°E–160°W)^18^.

The Niño3 and Niño3.4 indices are defined as the average SSTA in the regions of (5°S–5°N, 150°W–90°W) and (5°S–5°N, 170°W–120°W), respectively, using the ocean surface layer temperature of GODAS. Monthly mean zonal wind stress and 6-hourly mean zonal wind at 1000 hPa are from the NCEP-DOE (R2) reanalysis on 1° × 1° grid^32^. All the data are for Jan 1979–Dec 2016. The anomalies are referred to as the departures from monthly climatologies during Jan 1979–Dec 2016.

To display the time scale dependent variability of the Niño3.4 and WWV indices, as well as change in their frequency, a wavelet analysis is applied^15, 16^. To examine the change of zonal wind stress with respect to the SST variability, we compute the so-called Bjerknes feedback, a key process for ENSO growth or decay, which is defined as the regressions of zonal wind stress anomalies onto the Niño3 index following Lloyd *et al*.^19^, to measure the remote zonal wind response to a given SST change. The regression coefficient represents the coupling strength between the wind stress and Niño3 SSTAs^19^. Strictly speaking, the Bjerknes feedback denotes the zonal winds response to SSTs as well as their retroaction (favoring either the growth or dissipation of SST anomalies).

## Electronic supplementary material

Supplementary

## References

[1] National Research Council. *Assessment of Intraseasonal to Interannual Climate Prediction and Predictability*, 192 PP., ISBN-10: 0-309-15183-X, the National Academies Press, Washington, DC, USA (2010).

[2] Wang W, Chen M, Kumar A (2010). An assessment of the CFS real-time seasonal forecasts. Wea. Forecasting.

[3] Wang B (2009). Advance and prospectus of seasonal prediction: assessment of the APCC/CliPAS 14-model ensemble retrospective seasonal prediction (1980–2004). Clim. Dyn..

[4] Graham R (2011). Long-range forecasting and the Global Framework for Climate Services. Clim. Research.

[5] Barnston AG (2012). Skill of real-time seasonal ENSO model predictions during 2002–2011 — Is our capability increasing?. Bull. Amer. Meteor. Soc..

[6] Kumar A, Chen M, Xue Y, Behringer D (2015). An analysis of the temporal evaluation of ENSO prediction skill in the context of equatorial Pacific Ocean observing system. Mon. Weather Rev..

[7] McPhaden MJ (2012). A 21st century shift in the relationship between ENSO SST and warm water volume anomalies. Geophys. Res. Lett..

[8] Hu Z-Z (2013). Weakened interannual variability in the tropical Pacific Ocean since 2000. J. Clim..

[9] Hu Z-Z, Kumar A, Huang B, Zhu J, Ren H-L (2017). Interdecadal variations of ENSO around 1999/2000. J. Meteor. Res..

[10] Kumar A, Hu Z-Z (2014). Interannual and interdecadal variability of ocean temperature along the equatorial Pacific in conjunction with ENSO. Clim. Dyn..

[11] Hu Z-Z, Kumar A, Huang B (2016). Spatial distribution and the interdecadal change of leading modes of heat budget of the mixed-layer in the tropical Pacific and the association with ENSO. Clim. Dyn..

[12] Horii T, Ueki I, Hanawa K (2012). Breakdown of ENSO predictors in the 2000s: Decadal changes of recharge/discharge–SST phase relation and atmospheric intraseasonal forcing. Geophys. Res. Lett..

[13] Lübbecke JF, McPhaden MJ (2014). Assessing the 21st century shift in ENSO variability in terms of the Bjerknes stability index. J. Clim..

[14] An S-I, Wang B (2000). Interdecadal change of the structure of the ENSO mode and its impact on the ENSO frequency. J. Clim..

[15] Meyers SD, Kelly BG, O’Brien JJ (1993). An introduction to wavelet analysis in oceanography and meteorology: With application to the dispersion of Yanai waves. Mon. Weather Rev..

[16] Hu Z-Z, Nitta T (1996). Wavelet analysis of summer rainfall over North China and India and SOI using 1891–1992 data. J. Meteor. Soc. Japan.

[17] Meinen CS, McPhaden MJ (2000). Observations of warm water volume changes in the equatorial Pacific and their relationship to El Niño and La Niña. J. Clim..

[18] Thual S, Dewitte B, Ayoub N, Thual O (2013). An asymptotic expansion for the recharge-discharge model of ENSO. J. Phys. Oceanogr..

[19] Lloyd J, Guilyardi E, Weller H, Slingo J (2009). The role of atmosphere feedbacks during ENSO in the CMIP3 models. Atmos. Sci. Lett..

[20] Wittenberg AT, Rosati A, Lau N-C, Ploshay JJ (2006). GFDL’s CM2 global coupled climate models, Part III: Tropical Pacific climate and ENSO. J. Clim..

[21] Tseng Y-H, Hu Z-Z, Ding R-Q, Chen H-C (2017). An ENSO prediction approach based on ocean conditions. Clim. Dyn..

[22] Ding R (2017). Joint impact of North and South Pacific extratropical atmosphere variability on the onset of ENSO events. J. Geophys. Res. (Atmosphere).

[23] Hu Z-Z (2012). An analysis of warm pool and cold tongue El Niños: Air–sea coupling processes, global influences, and recent trends. Clim. Dyn..

[24] Bunge L, Clarke AJ (2014). On the warm water volume and its changing relationship with ENSO. J. Phys. Oceanogr..

[25] Ren H-L, Jin F-F (2013). Recharge oscillator mechanisms in two types of ENSO. J. Clim..

[26] Ren H-L, Jin F-F (2011). Niño indices for two types of ENSO. Geophys. Res. Lett..

[27] Yeh S (2009). El Niño in a changing climate. Nature.

[28] L’Heureux M, Collins D, Hu Z-Z (2013). Linear trends in sea surface temperature of the tropical Pacific Ocean and implications for the El Niño-Southern Oscillation. Clim. Dyn..

[29] Zhu J (2016). The role of off-equatorial surface temperature anomalies in the 2014 El Niño prediction. Sci. Rep..

[30] Kug J-S, Kang I-S, An S-I (2003). Symmetric and antisymmetric mass exchanges between the equatorial and off-equatorial Pacific associated with ENSO. J. Geophys. Res..

[31] Behringer, D. W. The Global Ocean Data Assimilation System (GODAS) at NCEP. Preprints, 11th Symp. on Integrated Observing and Assimilation Systems for Atmosphere, Oceans, and Land Surface, San Antonio, TX, Amer. Meteor. Soc., 3.3 [Available online at http://ams.confex.com/ams/87ANNUAL/techprogram/paper_119541.htm] (2007).

[32] Kanamitsu M (2002). NCEP-DOE AMIP-II Reanalysis (R-2). Bull. Amer. Meteor.

