# Supplementary material for: On the Shortening of the Lead Time of Ocean Warm Water Volume to ENSO SST Since 2000
Source: Sci Rep. 2017 Jun 27;7:4294. doi: 10.1038/s41598-017-04566-z (PMC5487327; doi:10.1038/s41598-017-04566-z)
Supplement: Supplementary file 1 — Supplementary [file 41598_2017_4566_MOESM1_ESM.pdf]

Supplementary Material of

"On the Shortening of the Lead Time of Ocean Warm Water Volume to ENSO SST Since 2000"

Zeng-Zhen Hu, Arun Kumar, Jieshun Zhu,
Bohua Huang, Yu-heng Tseng, and Xiaochun Wang

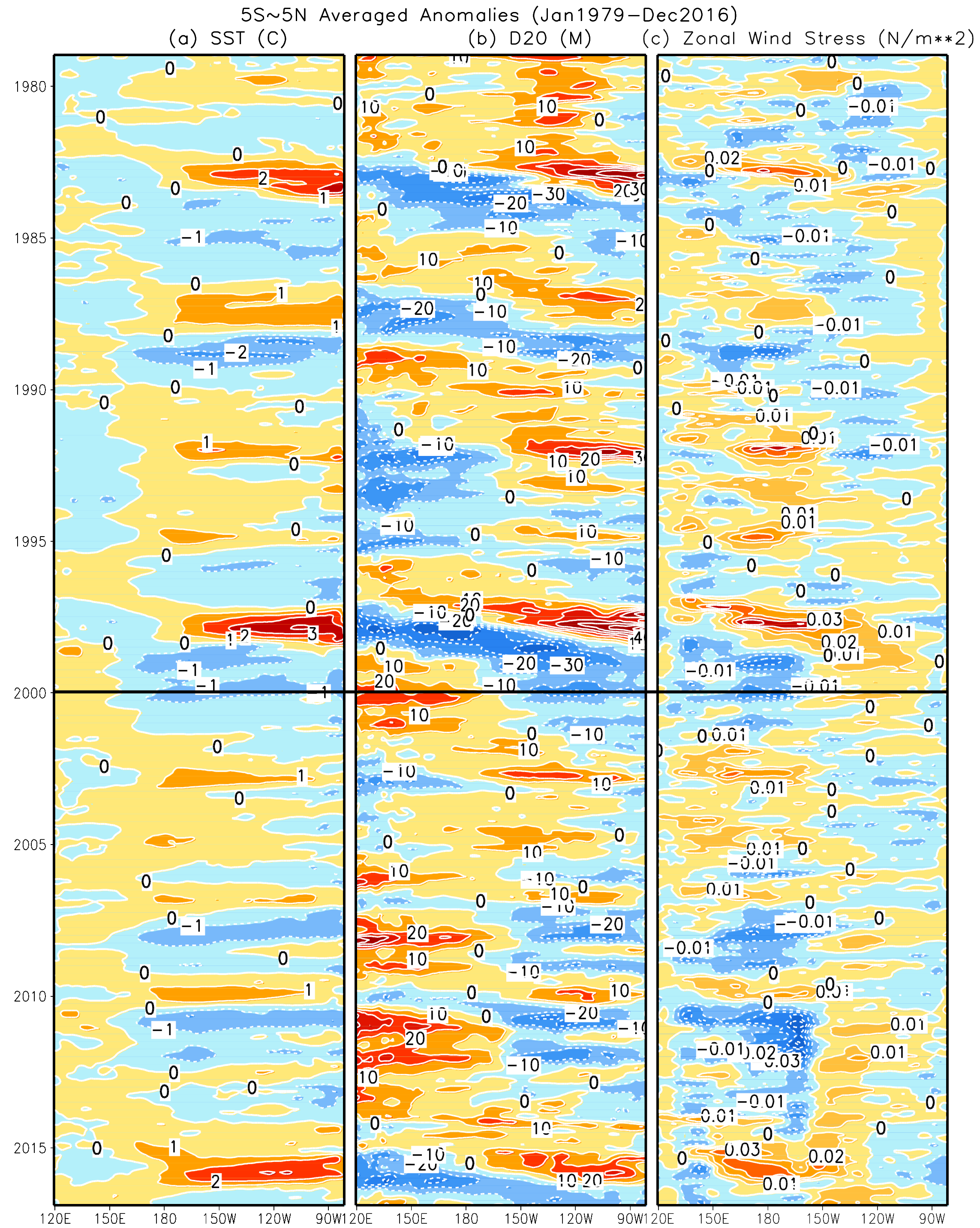

Supplementary Fig. S1: Time and longitude evolution of (a) SST (unit: ^{o}C), (b) D20 (unit: meter), and (c) zonal wind stress (unit: N/m^2) anomalies averaged in $5^{o}S$-$5^{o}N$ during Jan 1979-Dec 2016. Figure is generated by GrADS (http://cola.gmu.edu/grads/).

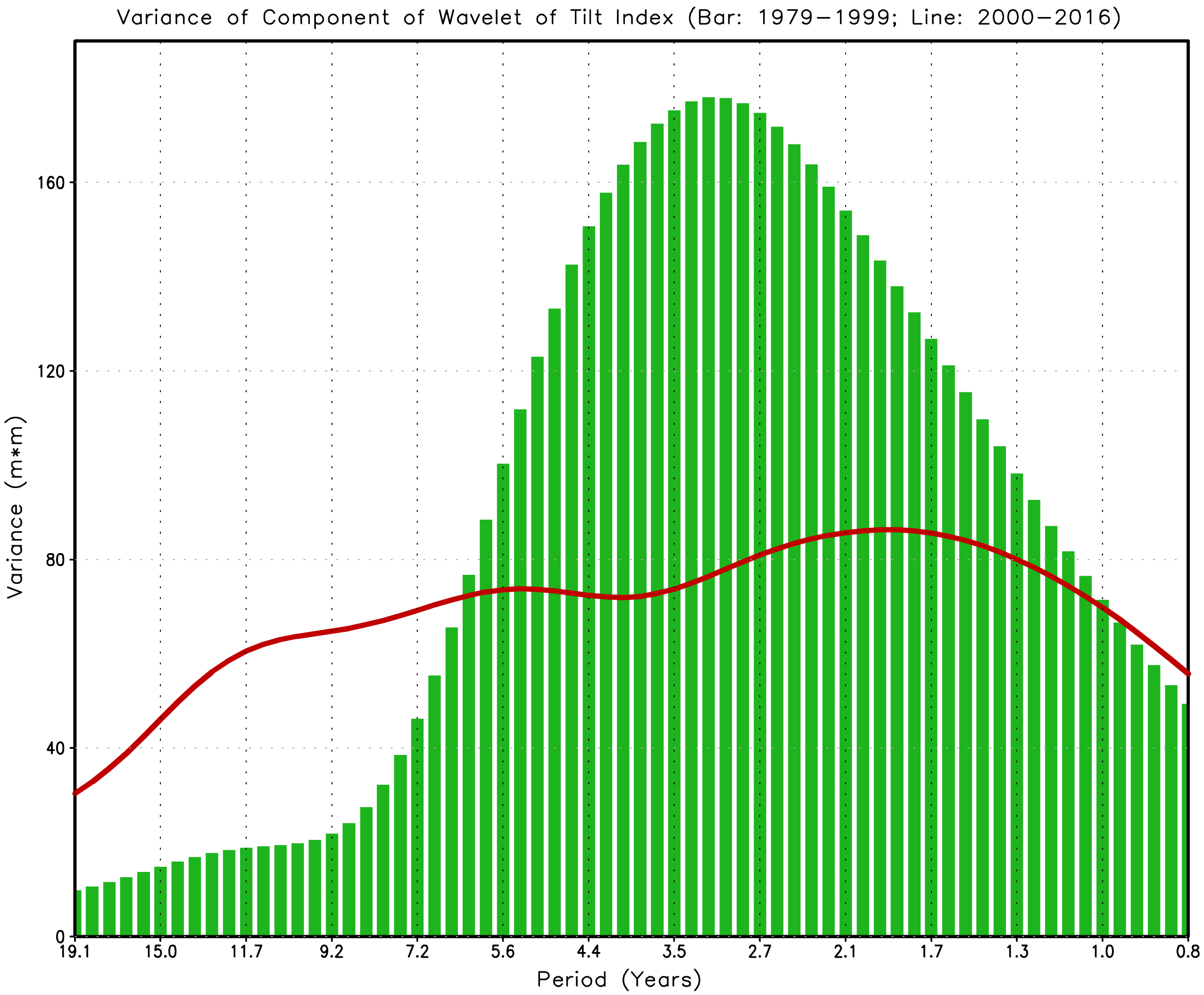

Supplementary Fig. S2: Variance dependence on time scales of the tilt index for the average in Jan 1979-Dec 1999 (bar) and Jan 2000-Dec 2016 (curve), based on the time scale decomposition of wavelet. The tilt index is defined as the difference of D20 between the average in (5°S-5°N, 160°-80°W) and (5°S-5°N, 120°E-160°W) (Thual et al. 2013). See text for the details of the wavelet calculation. Figure is generated by GrADS (http://cola.gmu.edu/grads/).

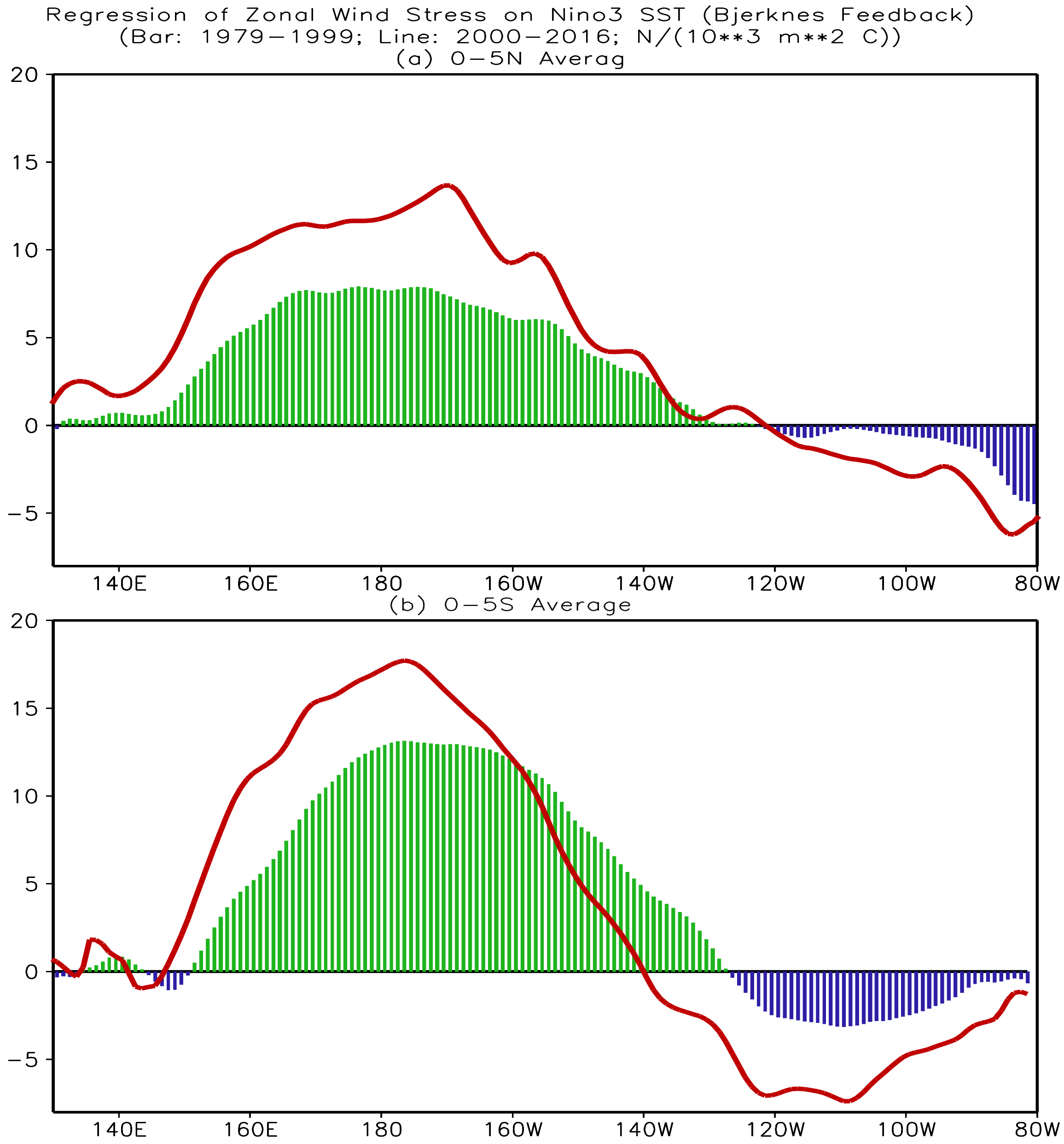

Supplementary Fig. S3: Simultaneous regressions of zonal wind stress anomalies onto the Niño3 index, which was referred to as the Bjerknes feedback, averaged in (a) 0°-5^oN, and (b) for 5^oS-0° and in 1979-99 (bar) and 2000-16 (curve). Figure is generated by GrADS (http://cola.gmu.edu/grads/).

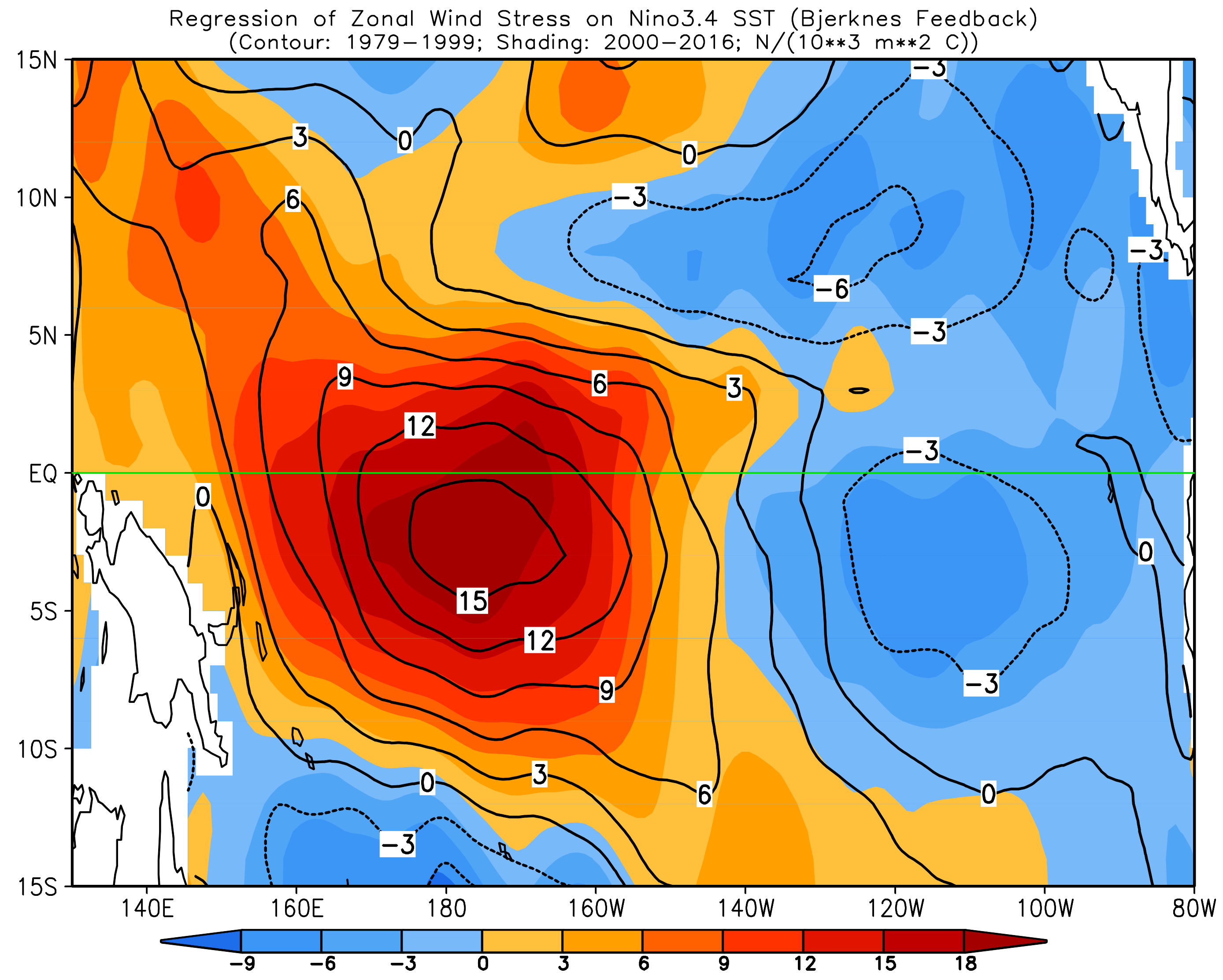

Supplementary Fig. S4: Simultaneous regressions of zonal wind stress anomalies onto the Niño3.4 index. Contours are for the regressions in Jan 1979-Dec 1999, and shadings for Jan 2000-Dec 2016. The unit is N/(10^3 m^2 ^oC) and the contour interval is 3. Figure is generated by GrADS (http://cola.gmu.edu/grads/).

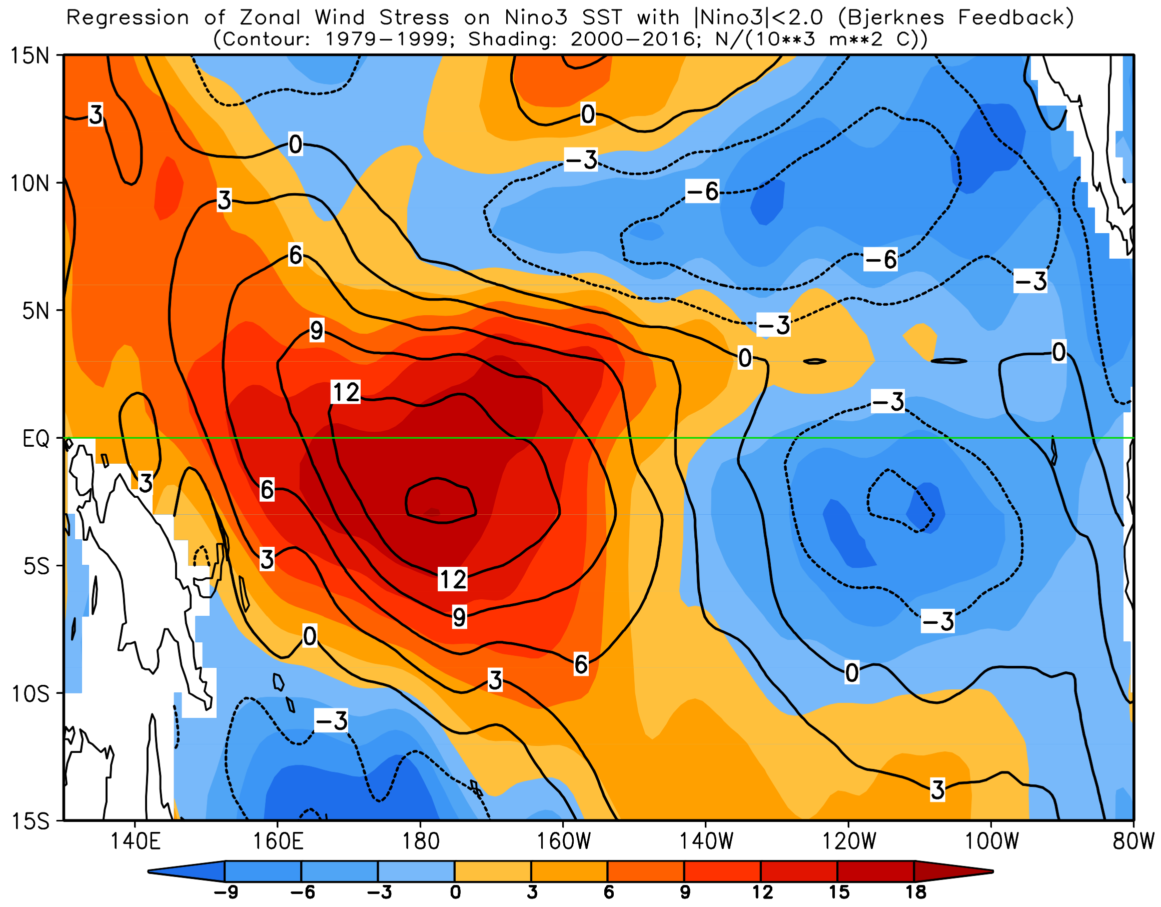

Supplementary Fig. S5: Simultaneous regressions of zonal wind stress anomalies onto the Niño3 index, which was referred to as the Bjerknes feedback. To exclude the extreme events, the regressions are computed only with absolute value of Niño3 index less than 2.0^{o}C. Contours (shadings) are for the regressions in Jan 1979-Dec 1999 (Jan 2000-Dec 2016) and Jan 1979-Dec 1999. The unit is N/(10^{3} m^{2} ^{o}C) and the contour interval is 3. Figure is generated by GrADS (http://cola.gmu.edu/grads/).
